# Measuring Temporal Instability of Momentary Affect, Subjective Health and Symptom Burden Depending on Environmental Parameters—An Ambulatory Assessment Study in Persons with Hay Fever

**DOI:** 10.3390/ijerph18020406

**Published:** 2021-01-06

**Authors:** Tim Rostalski, Silke Schmidt, Holger Muehlan

**Affiliations:** Department Health & Prevention, University of Greifswald, Robert-Blum-Street 13, D-17487 Greifswald, Germany; tim.rostalski@uni-greifswald.de (T.R.); silke.schmidt@uni-greifswald.de (S.S.)

**Keywords:** environmental parameters, allergic reactions, hay fever, quality of life, symptom burden, subjective health, momentary assessment, temporal instability

## Abstract

Our ambulatory assessment study explores the impact of the weather on the mental well-being of people with increased susceptibility. Participants with hay fever (*n* = 28) were assessed three times a day over a period of two weeks. Self-reported assessments covered different indicators of mental well-being, including momentary affect, subjective health as well as symptom burden. Based on tracked time stamps and location information, the data was matched with concurrent observation data from weather stations. We applied multilevel analysis to identify the main effects of selected environmental parameters (temperature, precipitation, wind power, sunshine duration and relative humidity) on all indicators of subjective well-being. Results confirm the main effects of sunshine duration, relative humidity and temperature on momentary affect as well as of sunshine duration, relative humidity and precipitation on subjective health and symptom burden. However, influences of environmental parameters on momentary affect were quite small and do not differ from effects documented in previous research in healthy samples with non-increased susceptibility.

## 1. Introduction

People’s momentary affect can vary from day to day or even during the course of a day, and is influenced by a variety of situational and environmental factors [1]. The weather is commonly believed to be an environmental factor that has an influence on affective experience, with people feeling happier on warm and sunny days than on cold and rainy days. Beside general approaches to link environmental parameters with mental health and well-being, such as the “ecosystem service perspective” [2], a multitude of specific studies have shown that temperature [3,4,5,6,7], sunlight [3,4,6,7], wind power [3], relative humidity [4,7] and rain [6,8] are associated with momentary affect. However, these effects tend to be small and inconsistent, and other studies have found no significant effects of weather on people’s moods [1]. Denissen et al. [3] proposed that this inconsistency may be caused by individual differences in weather dependencies. Klimstra et al. [6] tried to identify individual response classes and established four different weather reactivity types, called “Summer Lovers”, “Unaffected”, “Rain Haters” and “Summer Haters”, which differ in their affective responses to the weather. Of these four types, the “Summer Haters” showed an unexpected reactivity, reporting worse mood with higher temperatures, more sun and less precipitation, thus reacting to the weather in the opposite way to “Summer Lovers”. Klimstra et al. [6] mentioned the similarity to the opposing pattern of the two subtypes of seasonal affective disorder (SAD): Winter SAD and summer SAD.

An alternative explanation for the reaction style of “Summer Haters” could be due to seasonal allergic rhinitis, which is an inflammation of the nasal airways caused by pollen [9]. Characteristic symptoms are a running or blocked nose, itching of the nose, eyes, throat or ears and the urge to sneeze. Moderate to severe symptoms often lead to sleep disturbance, impairment of daily activities, sport and leisure and impairment of school or work [9]. As rhinitis mainly occurs during the pollen season, it is strongly related to weather. Weather conditions influence the amount of pollen in the air and their distribution. Temperature has been shown to have a positive effect on the occurrence of pollen allergens in the atmosphere, and relative humidity a negative effect [10,11]. Higher temperatures also increase pollen allergenicity [12]. The intensity of pollen allergens directly influences the symptomatology.

Despite its severe effects, very few studies have been published on the impact of seasonal allergic rhinitis on well-being and momentary affect. The available studies show an association of allergic rhinitis with reduced quality of life [13,14,15] or a lower positive affect [16,17] and increased levels of tiredness [14]. The available studies that investigate the relationship between weather and affective states in seasonal rhinitis focus on the impaired well-being or quality of life [14,18], thus viewing the allergy from a more clinical perspective. In contrast, the present study investigates seasonal rhinitis and the dependencies between weather and affective states from a nonclinical perspective. People with seasonal rhinitis are treated as a population with high sensitivity for weather in terms of fears and avoidance of weather conditions supporting pollen allergenicity. As this behavior strongly resembles that of “Summer Haters”, it is suggested that dependencies between weather and affective states are likely to occur in both healthy people described as “Summer Haters” and people with allergic rhinitis in a similar way.

The main aim of the present study is to examine the associations of weather and mood, and of symptom experience and quality of life, in more detail. In particular, the effects of several objective weather parameters on different dimensions of momentary affect (valence, alertness, calmness) are investigated, as well as various areas of symptom reports and subjective health reports in a population with high sensitivity for weather.

## 2. Materials and Methods

### 2.1. Sample Characteristics

The sample consisted of 28 participants (18 women and 10 men) with seasonal allergic rhinitis. Within this sample, three participants stated that they were generally affected very much by the rhinitis, 11 participants stated they were fairly affected, five participants were moderately affected and nine were just slightly affected. All participants were recruited through email advertising using the university mailing list (25 students and three university employees). Students came from different faculties of the university. The mean age was 24.04 (SD = 2.53) years, ranging from 20 to 30 years. All participants gave their informed consent. Data was collected during the grass pollen season in July and August.

### 2.2. Study Procedure

The participants first completed a questionnaire on their impairment by seasonal allergic rhinitis and the presence of other allergies. Then they were equipped with an iPod Touch (Apple Inc., Cupertino, CA, USA). The ambulatory assessment procedure was implemented using iDialogPad (Gerhard Mutz, University of Cologne, Cologne, Germany). Participants were told that, for the next 14 days, they would be acoustically prompted three times a day, at around 10 a.m., 4 p.m. and 10 p.m. (with random time windows of ±10 min) to answer questions on their iPod. The time points were chosen to represent the three parts of one day (morning, afternoon, evening). After practicing the procedure, during which they had the opportunity to ask questions, the participants signed an informed consent form for participating in this study and received instructions about the ambulatory assessment procedure and contact information in case of technical problems.

A second session in the laboratory took place two weeks later. Participants returned the iPods, completed a questionnaire and evaluated the ambulatory assessment procedure and the usability of the iPod. Then they were informed about the purpose of the study.

### 2.3. Ambulatory Assessment

At each of the three measurement points, participants filled out a questionnaire on their momentary affect, symptoms of seasonal allergic rhinitis and momentary self-rated health status. Furthermore, they had to supply the postcode and name of the town or village where they were at the moment of assessment:

(a) Momentary affect: Momentary affect was assessed with a short form of the Multidimensional Mood State Questionnaire (MDMQ) [19]. The German version of the MDMQ consists of 12 items, rated on a 5-point Likert scale (ranging from “1—not at all” to “5—extremely”) and three subscales: Valence (good-bad), alertness (awake-tired) and calmness (calm-nervous). Four items are assigned to each scale, with two items assessing positive affect states and two items assessing negative affect states. Sum scores were computed for the three subscales (with items assessing negative affect states reversed scored). Valence is measured with the items “content”, “great”, “bad” and “uncomfortable”; alertness is assessed according to the items “rested”, “energetic”, “worn-out” and “tired”; and calmness according to “composed”, “relaxed”, “restless” and “uneasy”. Each item is preceded by the sentence ‘‘Right now I feel...’’. All items appeared in the original order of the MDMQ at each trial. Cronbach’s αs of the three scales range from 0.73 to 0.89 over four repeated measures [19].
(b) Seasonal allergic rhinitis: Symptoms of seasonal allergic rhinitis were assessed with symptom scales from the Allergen Challenge Chamber—Allergic Rhinitis Quality of Life Questionnaire [20]. This questionnaire is designed to measure impairments through rhinitis symptoms in an acute situation. The items are rated on a 7-point Likert scale (from “0—no impairment” to “7—very strong impairment”). Participants were asked how much they are impaired at this moment by the following symptoms: (1) Nose symptoms, which are measured with the items “runny nose”, “blocked nose”, “itching nose” and “urge to sneeze”; (2) eye symptoms, measured with the items “runny eyes”, “itchy eyes” and “red eyes”; and (3) other symptoms, represented by the items “tickle in the throat”, “itchy throat”, “itchy palate” and “itchy ears”. Cronbach’s αs of the three scales range from 0.87 to 0.90 [20].
(c) Self-rated health status (SRHS): To assess the general within-day variation of participants’ health, the momentary self-rated health status was measured with the question “How is your health at this moment?” To detect even small fluctuations over the day, a Visual Analogue Scale (VAS) with a length of 101 points was used. The anchors were “0—very poor” on the left end and “100—very good” on the right end.
(d) Compliance: For all participants, the number of completed questionnaires was 1108 of 1176 possible questionnaires (28 participants × 14 days × 3 time points per day). This is a response rate of 94.2%, which is fairly good for an ambulatory assessment study. The number of completed reports per participant ranged from 31 to 42 of 42 possible reports (M = 39.57, SD = 3.19). The data indicates a high compliance with the procedure. Among the 1108 completed questionnaires, there was no missing data.

### 2.4. Weather Data

The weather parameters were obtained from the German Weather Institute (Deutscher Wetterdienst, Offenbach/Main, Germany; http://www.dwd.de). The postcodes supplied by the participants were used to match the ambulatory assessment data with the weather variables. Forty-four different postcodes were reported by the participants. To find the nearest weather station for every postcode, the orthodromic distances between the reporting locations and the weather stations were calculated. To obtain the geographic coordinates (latitude and longitude) for every unique postcode, data from OpenGeoDB (http://www.opengeodb.org) were used. The geographic coordinates of the 81 weather stations with hourly weather parameters were obtained from the German Weather Institute. All together 18 weather stations were relevant for this study, with a mean distance of 26.46 km (16.5 miles) and a range from 1.53 km to 62.19 km (0.95 mi to 38.64 mi) to the postcodes provided by the participants. For these stations the weather data on temperature (°C), relative humidity (%), precipitation (mm), wind power (m/s) and sunshine duration (min) for the hour before each self-report record were obtained. For 23 time points, not all weather parameters were available, which were thus taken out of the analysis. This resulted in a final dataset of 1085 completed ambulatory assessment records with matching weather parameters.

### 2.5. Data Analyses

Because of repeated measurements within a single individual and the frequent occurrence of missing records, it is recommended to use a multilevel analysis for analyzing ambulatory assessment data [21,22,23]. Moreover, multi-level approaches had already been used to analyze data linking environmental and individual variables in previous research [3,24]. To test the impact of weather variables on momentary affect, two-level linear-mixed regression models were computed for the three dependent variables valence, alertness and calmness. First, an unconditional model was calculated to assess the total variance at each level. Next, the weather parameters were entered in the model as simultaneous predictors, where both intercepts and slopes were allowed to vary across persons. Then the proportion of explained variance within person was estimated [25].

The same procedure was used to test the impact of weather variables on symptom report and health. First, an unconditional model was calculated for every symptom scale and the SRHS, after which the weather parameters were entered as predictors and the explained variance within person was calculated. For all analyses, STATA statistical software (version 11.2; Stata Corporation, College Station, TX, USA) was used with its mixed modelling tool (xtmixed). All weather parameters were grand mean centered, as recommended by Nezlek [23].

### 2.6. Compliance with Ethical Standards

All procedures performed in the studies involving human participants were in accordance with the ethical standards of the institutional and/or national research committee and with the 1964 Helsinki declaration and its later amendments or comparable ethical standards. No formal ethical approval for this study was necessary since no human tissue was gathered. Participants received verbal and written information about the study prior to giving consent. Informed and written consent was obtained from all participants upon the agreement that their identities would not be revealed, and that they could withdraw from participation at any time.

## 3. Results

### 3.1. Respondents

The average temperature over the 1085 time points was 18.39 °C (SD = 3.69), ranging from 10.9 °C to 32.9 °C. The average temperature in Germany from July to August for the last decade is 18.12 °C. The overall pollen exposure was slightly below average (−6%), also with respect to strong pollen exposure in particular (−12%), as compared to the average of the last decade in the study region. The descriptive statistics of the other weather parameters are given in Table 1. The associations (Pearson’s correlation coefficient) between the weather parameters ranged between |0.08| and |0.70| and were all significant, with the exception of the correlations between wind power and relative humidity (r = 0.05, *p* = 0.13), and sunshine duration (r = 0.05, *p* = 0.10). Table 1 also shows the descriptive statistics of momentary affect scales, symptom report and self-rated health status. The correlations between the three subscales of the MDMQ were high and ranged between 0.44 and 0.82.

### 3.2. Momentary Affect and Weather Parameters

Table 2 shows the relation between valence, alertness, calmness and different weather parameters. Few weather parameters were related to momentary affect. Only temperature had a significant effect on valence: With each unit rise in temperature, valence increased 0.065 units. The addition of temperature, relative humidity, precipitation, wind power and sunshine duration to the unconditional model explained 6.0% of the within-person variance of valence.

Alertness was also related to temperature (b = 0.105, *p* < 0.05) and mostly to sunshine duration (b = 0.017, *p* < 0.01). Relative humidity, precipitation and wind power were not associated with any changes in alertness. All five weather parameters together explained 5.7% of the within-person variance of alertness.

Regarding calmness, changes in temperature, relative humidity, precipitation, wind power and sunshine duration explained 4.6% of the within-person variance. Only relative humidity significantly predicted calmness. The rise of relative humidity by one unit increased the calmness score by 0.019 units. Temperature, precipitation, wind power and sunshine duration were not significantly related to calmness.

### 3.3. Symptom and Health Report and Weather Parameters

As can be seen in Table 3 below, not all five weather parameters contributed to the fluctuation of the seasonal allergic rhinitis symptoms and the general health ratings. Temperature and wind power were not significantly related to any of the outcome variables. Relative humidity and sunlight were negatively related to nose symptoms: With each unit rise in relative humidity, nose symptoms decreased by 0.046 units. With each unit increase in sunshine duration, nose symptoms were reduced by 0.024 units. Adding temperature, relative humidity, precipitation, wind power and sunshine duration to the unconditional model explained 8.6% of the within-person variance of nose symptoms.

The five weather parameters explained 4.7% of the within-person variance of eye symptoms, although none of the independent parameters significantly contributed to the prediction of eye symptoms.

Only sunshine duration was related to the other rhinitis symptoms. With each unit increase in sunshine duration, other symptoms decreased by 0.013 units. Temperature, relative humidity, precipitation, wind power and sunshine duration explained about 7.9% of the within-person variance of other rhinitis symptoms.

Finally, precipitation and sunlight duration contributed independently to the prediction of self-rated health status (SRHS). Precipitation was the weather variable with the greatest impact on SRHS (b = 1.137, *p* < 0.001). With each unit rise in sunlight duration, SRHS increased 0.097 units. Adding temperature, relative humidity, precipitation, wind power and sunshine duration as predictors of SRHS explained 8.6% of within-person variance.

## 4. Discussion

### 4.1. Main Findings and Comparison with Previous Research

This study investigated the effect of weather variables on momentary affect as well as symptom and health reports of people with allergic rhinitis. The results showed significant effects of weather on the three affect dimensions valence, alertness and calmness, where valence was only influenced by temperature. Moreover, significant positive effects of temperature and sunshine duration on alertness were found, implying that persons are more awake and less tired when the weather gets warmer and it’s sunnier outside. These results are in line with the negative effect of sunshine on tiredness reported by Denissen et al. [3]. The authors argue that less exposure to sunshine lowers the level of vitamin D, and therefore could be responsible for increases in tiredness. Relative humidity was found to predict greater calm, which agrees with the results of a recent study concerning the general population [26].

Intensity of allergen exposure directly influences symptomatology and frequently pollen concentrations decrease when there is precipitation [27,28]. Thus, pollen-sensitive individuals feel less burden of disease in terms of less symptomatology and higher subjective health when it rains. Precipitation was the weather variable with the greatest impact on SRHS (b = 1.137, *p* < 0.001).

To the weather parameters sunshine, temperature and precipitation used by Klimstra et al. [6], the parameters relative humidity and wind power were added, which have been shown in other studies to be relevant or which seem to be important for this population specifically, as they have an effect on the occurrence and the dispersal of pollen allergens [12,29]. Nevertheless, neither parameter had any main effect on mood, which agrees with the overall small influence of weather on mood in this population. The effect sizes for the impact of weather on mood are comparable to results reported from previous studies with non-specific populations [7]. In contrast, stronger associations were found between weather and symptom report. This surprising result could be explained by habituation to adaption levels in people with seasonal allergic rhinitis.

According to the Adaptation Level Theory [30], undesired events, such as the experience of allergic rhinitis symptoms, evoke negative mood and emotions if they deviate from the individual’s frame of reference. However, since the experience of these events shape the frames of reference over time, individuals adapt and the events no longer elicit negative affective states. Thus, recurring symptom experiences during the pollen season over the years could be the reason for the lack of association with momentary affect found in this study. Moreover, since study participants were allowed to use medication if needed to reduce symptoms, symptom burden might not have been long or intensive enough to cause substantial mood variations. Perhaps the mere awareness of potential self-medication in case of acute symptom burden could explain the lack of impact symptom experience has on momentary mood. 

The hypothesis that people with seasonal allergic rhinitis show similar associations between weather and mood as the so-called “Summer Haters” [6]—people whose mood worsens in case of more sun, higher temperatures and less precipitation—was not substantiated. In contrast, higher temperatures were associated with better mood for seasonal allergic rhinitis sufferers.

One of the strengths of this study is our approach to real-time data capture. So far, only a few studies applied ambulatory assessment or related methodological approaches (ecological momentary assessment, experience sampling method) to investigate the impact of environmental parameters in as sample of persons with hay fever [31,32,33]. Despite of the innovative approach of our study design, some restrictions still remain.

### 4.2. Limitations

A main limitation is the inclusion of self-selected participants with confirmed and presumed seasonal allergic rhinitis and reporting symptomatic at baseline. Further limitations of our study concern the fact that the time that participants spent outside was not taken into account, even though this is considered a very important moderator [4]. Time spent outdoors should therefore be considered in future studies. Furthermore, it is recommended to assess participants’ different past and current symptom experiences. Moreover, given the high variability of local weather even within very small areas, the relevance and validity of the weather data for our analyses is questionable and impaired, considering the average distance between participants and weather stations. However, these were the best data available. 

## 5. Conclusions

To sum up, our results indicate that weather has a strong influence on persons with seasonal allergic rhinitis. This effect is most evident on the level of symptom reports. The influence of weather on momentary mood, on the other hand, is quite small and different as was expected. With respect to weather parameters that support an increased pollen concentration, temperature has an influence on valence and alertness, whereas humidity has an influence on calmness only. This result points to the differential impact of weather parameters on mood, which also highlights the potential of a multidimensional mood measurement.

## Figures and Tables

**Table 1:** Descriptive statistics of environmental (weather) parameters, momentary affect, health status and symptom burden of seasonal allergic rhinitis.

	M	Min	Max	SD
**Weather parameters**				
Temperature (in °C)	18.39	10.9	32.9	3.69
Humidity (in %)	77.37	33.0	100.0	15.35
Precipitation (in mm)	0.35	0.0	15.9	1.35
Wind power (in m/s)	3.74	0.0	15.0	2.2
Sunshine (min)	13.62	0.0	60.0	20.77
**Momentary Affect**				
Valence	15.15	3	20	4.26
Alertness	12.62	3	20	3.7
Calmness	14.18	2	20	4.23
**Symptom Burden**				
Nose Symptoms	3.98	0	24	4.41
Eye Symptoms	1.61	0	15	2.75
Other Symptoms	1.56	0	16	2.87
**SRHS ^1^**	83.39	0	100	17.9

^1^ SRHS = self-rated health status.

**Table 2:** Relation between several domains of momentary affect (valence, alertness, calmness) and different environmental (weather) parameters.

	Valence		Alertness		Calmness	
	Coefficient	z-Ratio	Coefficient	z-Ratio	Coefficient	z-Ratio
Intercept	15.05	20.88	12.58	27.31	14.08	19.23
Temperature	0.065	2.07	0.105	2.41	0.037	ns
Humidity	0.016	ns	0.014	ns	0.019	2.64
Precipitation	−0.006	ns	0.108	ns	0.004	ns
Wind Power	−0.031	ns	0.02	ns	−0.024	ns
Sunshine	0.008	ns	0.017	2.72	0.007	ns

Notes: Fixed effects. ns = not significant (*p* < 0.05).

**Table 3:** Relation between seasonal allergic rhinitis symptoms, self-rated health status (SRHS) and different environmental (weather) parameters.

	Nose Symptoms		Eye Symptoms		Other Symptoms		SHRS ^1^	
	Coefficient	z-Ratio	Coefficient	z-Ratio	Coefficient	z-Ratio	Coefficient	z-Ratio
Intercept	4.088	7.66	1.652	4.17	1.557	4.70	83.02	36.51
Temperature	0.085	ns	0.025	ns	0.031	ns	−0.371	ns
Humidity	−0.046	−3.56	−0.011	ns	−0.014	ns	0.031	ns
Precipitation	−0.068	ns	−0.084	ns	−0.077	ns	1.137	3.45
Wind Power	0.021	ns	−0.009	ns	0.041	ns	−0.437	ns
Sunshine	−0.024	−3.02	−0.008	ns	−0.013	−2.58	0.097	3.21

^1^ SRHS = self-rated health status. Notes: Fixed effects. ns = not significant (*p* < 0.05).

## Data Availability

The data presented in this study are available on request from the corresponding author. The weather parameters could be obtained from the German Weather Institute (Deutscher Wetterdienst, Offenbach/Main, Germany; http://www.dwd.de).
